# Autonomic Dysfunction and Cardiac Performance in Pregnant Women with Hypertensive Disorders: A Comparative Study Using Heart Rate Variability and Global Longitudinal Strain

**DOI:** 10.3390/life14081039

**Published:** 2024-08-20

**Authors:** Carina Bogdan, Adrian Apostol, Viviana Mihaela Ivan, Oana Elena Sandu, Ion Petre, Izabella Petre, Luciana-Elena Marc, Felix-Mihai Maralescu, Daniel Florin Lighezan

**Affiliations:** 1 Department VII, Internal Medicine II, Discipline of Cardiology, “Victor Babeş” University of Medicine and Pharmacy, Eftimie Murgu Sq. No. 2, 300041 Timişoara, Romania; carina.bogdan@umft.ro (C.B.); ivan.viviana@umft.ro (V.M.I.); oana.ciolpan@umft.ro (O.E.S.); 2 Centre for Molecular Research in Nephrology and Vascular Disease, Faculty of Medicine “Victor Babeş”, 300041 Timișoara, Romania; marc.luciana@umft.ro (L.-E.M.); mihai.maralescu@umft.ro (F.-M.M.); 3 Department of Functional Sciences, Medical Informatics and Biostatistics Discipline, “Victor Babeş” University of Medicine and Pharmacy, Eftimie Murgu Sq. No. 2, 300041 Timişoara, Romania; petre.ion@umft.ro; 4 Department of Obstetrics and Gynecology, “Victor Babes” University of Medicine and Pharmacy Timișoara, 300041 Timișoara, Romania; petre.izabella@umft.ro; 5 Department of Internal Medicine II, Discipline of Nephrology, “Victor Babeș” University of Medicine and Pharmacy, 300041 Timișoara, Romania; 6 Center of Advanced Research in Cardiology and Hemostaseology, University of Medicine and Pharmacy “Victor Babes” Timisoara, E. Murgu Square, Nr. 2, 300041 Timișoara, Romania; dlighezan@umft.ro; 7 Department V, Internal Medicine I, Discipline of Medical Semiology I, “Victor Babeș” University of Medicine and Pharmacy, 300041 Timișoara, Romania

**Keywords:** heart rate variability, autonomic dysfunction, cardiac performance, global longitudinal strain, hypertensive disorders of pregnancy, preeclampsia

## Abstract

(1) Background: Pregnancy induces significant physiological adaptations with substantial impacts on the cardiovascular system. Hypertensive disorders of pregnancy (HDPs) are connected to significant risks of maternal and fetal complications, contributing significantly to morbidity and mortality across the globe. This study focuses on evaluating autonomic dysfunction by analyzing heart rate variability (HRV) and assessing cardiac performance through global longitudinal strain (GLS) using speckle tracking echocardiography, as well as examining diastolic function in pregnant women with HDP compared to healthy pregnant controls. (2) Methods: A case–control study was conducted involving pregnant women diagnosed with gestational hypertension (GH), preeclampsia (PE), or severe preeclampsia (SPE) as the case group, and healthy pregnant women as the control group. HRV was measured to evaluate autonomic function, GLS was assessed using speckle tracking echocardiography, and diastolic function was evaluated through standard echocardiographic parameters. Data were analyzed to compare cardiac performance and autonomic regulation between the HDP and control group, as well as among the different HDP subgroups. (3) Results: The HDP group exhibited significantly reduced HRV parameters compared to healthy controls, indicating notable autonomic dysfunction. Speckle tracking echocardiography revealed lower GLS among women with HDP, particularly in those with SPE, compared to the control group. Diastolic dysfunction was also present in the HDP group. (4) Conclusions: HRV and GLS are valuable non-invasive tools for detecting autonomic dysfunction and cardiac performance impairments in pregnant women with hypertensive disorders. These findings suggest that autonomic and cardiac dysfunctions are prevalent in HDP.

## 1. Introduction

Pregnancy involves significant adaptive changes in the body, placing a substantial burden on the cardiovascular system. During a typical pregnancy, the cardiovascular system changes in multiple aspects to ensure adequate blood flow to the uterus and placenta, which is essential for delivering oxygen and nutrients to the fetus [1]. A key component in managing these pregnancy-related changes is the autonomic nervous system (ANS), which regulates the interaction of various essential systems in a complex manner [2,3].

The sympathetic nervous system (SNS) and the parasympathetic nervous system (PNS), which are two functional components of the ANS, work together to mediate the body’s hemodynamic adjustments during pregnancy [4].

Hypertensive disorders of pregnancy (HDPs) are prevalent worldwide and associated with higher rates of morbidity and mortality in both mothers and fetuses [5,6,7]. They encompass chronic pre-existing hypertension, gestational hypertension (GH), and preeclampsia (PE) [6]. 

The SNS is essential for meeting the energetic requirements of the organism during times of stress or demand. This system facilitates adaptive responses during pregnancy. In particular, in cases of PE, SNS activity becomes significantly elevated, both before the onset and during the progression of maternal hypertension. This increased activity reflects the body’s effort to cope with the altered physiological state and the additional demands placed on the cardiovascular system by pregnancy [8]. 

While evidence indicates that placental ischemia and subsequent vascular dysfunction contribute to hypertension in PE, the specific role of the sympathetic division of the ANS in the two-stage theory mediating the pathogenesis of this maternal disorder remains less understood [9,10,11].

Cardiac dysautonomia, characterized by the malfunction of the ANS in regulating cardiovascular homeostasis, can lead to a wide array of symptoms and complications. This dysfunction negatively impacts cardiovascular health, increasing the risk of cardiovascular disease.

Heart rate variability (HRV) is a valuable measure for assessing how well the ANS regulates cardiovascular function. It provides insight into the intricate interaction between the sympathetic and parasympathetic branches of the ANS. HRV is commonly employed as a prognostic indicator for cardiac diseases and is currently used to assess the effects of autonomic imbalance on various conditions [8,12]. 

HRV assesses the changes in the intervals of time between consecutive heartbeats. Analyzing HRV in both time and frequency domains, one can gauge the balance between the sympathetic and parasympathetic effects on heart function, providing a window into autonomic control. Reduced HRV has been shown to independently predict higher mortality rates and serve as a risk factor for sudden death, indicating autonomic dysregulation [13].

Left ventricular (LV) function is widely recognized as a key indicator of long-term survival for individuals with various cardiac conditions. Among the different methods used to assess LV systolic performance, LV ejection fraction (LVEF) is the most commonly employed echocardiographic measure [14]. 

A relatively recent advancement for evaluating LV function is speckle tracking imaging, which offers high temporal and spatial resolution for assessing myocardial strain [15,16]. 

Global longitudinal strain (GLS), which measures the degree of myocardial deformation, has emerged as a crucial tool for assessing LV function in clinical practice. GLS provides greater sensitivity and reproducibility in identifying subclinical cardiac alterations compared to traditional methods. Studies suggest that, during pregnancy, the use of speckle tracking imaging, instead of conventional echocardiography, better identifies the subclinical changes in LV function. Women with PE exhibit significant reductions in GLS when compared to pregnant patients without HDP [17,18]. 

Our study, which aims to examine HRV and cardiac performance during pregnancy, could offer new insights into the relationship between HDP and the regulation of the ANS as well as myocardial changes. This study highlights the link between HDP and decreased HRV, emphasizing autonomic dysfunction as a significant feature of these conditions. HRV, reflecting autonomic imbalance, could represent a non-invasive means of evaluating the extent of autonomic disturbances during pregnancy. Additionally, with further investigation, HRV might prove valuable as a predictive tool for assessing the burden of cardiovascular complications related to HDP. 

## 2. Materials and Methods

### 2.1. Study Population

Our research was a case–control study. Cases were pregnant women diagnosed with GH or PE with variable degrees of severity, while controls were healthy pregnant women. The inclusion criterion stated that both cases and controls had viable pregnancies with gestational age above 20 weeks calculated from the first day of the last menstrual period. 

Between January 2022 and December 2023, patients were referred to the Cardiology Department for a comprehensive cardiovascular evaluation. None of these patients had any prior history of cardiovascular disease or were on any medications. Each patient received an extensive clinical assessment that involved an in-depth medical history and physical examination. Data on demographic characteristics, medical history, and biological workout were collected.

According to the American College of Obstetricians and Gynecologists (ACOG) [5,19,20], a diagnosis of hypertension during pregnancy is made if the systolic blood pressure (SBP) is 140 mmHg or higher, diastolic blood pressure (DBP) is 90 mmHg or higher, or both. This diagnosis should ideally be confirmed on two separate occasions, or at least 4 h apart. GH is characterized by new-onset hypertension after 20 weeks of gestation, without the features of PE, and typically resolves postpartum with BP returning to normal. PE is defined by the presence of hypertension and either proteinuria or, in the absence of proteinuria, new-onset hypertension accompanied by other signs of maternal organ dysfunction. Proteinuria is diagnosed if a 24 h urine collection shows at least 300 mg of protein, if the protein-to-creatinine ratio in a single urine sample is 0.3 or greater, or if a dipstick reading is 2+ in the absence of other quantitative methods [5,19,20].

SPE is diagnosed when PE is accompanied by severe hypertension (SBP exceeding 160 mmHg, DBP exceeding 110 mmHg, or both), or by at least one of the following: thrombocytopenia, impaired liver function without an alternative cause, worsening renal function, pulmonary edema, or severe neurological symptoms such as new, unresponsive headaches or visual disturbances without another explanation [5,19,20]. In our study, SPE was defined as PE with an SBP greater than 160 mmHg, DBP greater than 110 mmHg, or both. 

Patients with a history of certain conditions were not included in this study. These excluded conditions comprised arterial hypertension, heart valve disorders, ischemic heart disease, bradyarrhythmias or tachyarrhythmias, atrial fibrillation, conduction disturbances such as branch blocks, metabolic disorders such as diabetes, cerebrovascular disease, chronic pulmonary disease, liver or renal insufficiency, malignancy, thyroid disorders, and those on medications impacting heart rate or conduction [21]. 

### 2.2. Heart Rate Variability Analysis and Cardiac Performance Evaluation

Each patient underwent 24 h ambulatory electrocardiographic (ECG) monitoring using a Holter monitor. The ECG recordings were reviewed to evaluate HRV, detect arrhythmias, and identify any electrical irregularities during both daily activities and sleep. To minimize the likelihood of artifacts in the recordings, patients were instructed to avoid vigorous physical activities. The Holter monitor data were transferred to a computer and processed using Labtech Cardiospy software. Each recording was visually inspected, with artifacts being manually edited out to ensure that at least 20 h of usable data were retained after the removal of interferences [21]. 

The HRV parameters were extracted using Labtech Cardiospy v5.03 software. These parameters were selected based on the guidelines from the European Society of Cardiology and the North American Society of Pacemaker and Electrophysiology, and analyzed using linear methods [22]. The linear analysis techniques applied for HRV measurement included both time and frequency domain analyses. In the time domain, the parameters assessed were RR intervals (or NN intervals), the standard deviation of RR intervals (SDNN), the root mean square of successive RR interval differences (RMSSD), and the percentage of adjacent NN intervals differing by more than 50 milliseconds (pNN50). For the frequency domain analysis, parameters included total power (TP), low frequency (LF, 0.04 to 0.15 Hz), and high frequency (HF, 0.15 to 0.4 Hz). The LF and HF components were calculated using either the fast Fourier transform algorithm or autoregressive modeling techniques. 

Echocardiographic assessments were conducted using the ESAOTE MyLabX8 Platform ultrasound systems with a 1–5 MHz transducer. Participants underwent a comprehensive evaluation that included both conventional 2-dimensional echocardiography and color Tissue Doppler Imaging (TDI) to analyze cardiac structure and function, focusing on both diastolic and systolic performance [21]. 

Doppler Echocardiography was employed to measure mitral inflow velocities and evaluate diastolic function. Using pulsed-wave Doppler from the apical view, peak velocities of early diastolic (E) and atrial (A) filling phases were recorded, along with the deceleration time of the E-wave. These parameters were used to calculate the E/A ratio.

TDI further assessed diastolic function by measuring peak early diastolic longitudinal velocity (E′) at the mitral annular segments (septal and lateral) in the four-chamber view. An average E′ value was calculated to determine the E/E′ ratio. Diastolic dysfunction type was defined according to the ASE/EACVI Guidelines and Standards [23]. For the LVEF assessment the method for obtaining LV volumes is the standard Simpson’s Bi-plane [23,24]. All patients in the study had an LVEF greater than 50%. 

GLS was measured using two-dimensional speckle tracking echocardiography in the apical 2-chamber, 3-chamber, and 4-chamber views. Initially, an automated function was used to trace the endocardial border of the LV at end-systole. This automated tracing was then visually inspected and adjusted manually, if necessary, to ensure accurate tracking of the myocardial speckles. GLS was calculated as the average peak strain derived from the three apical views, with the region of interest set to encompass the entire LV. In cases where speckle tracking could not be achieved in one or more of the chamber views, the GLS was computed using the available views and averaged to provide the final measurement [24]. 

### 2.3. Statistical Analysis

To characterize the study population, descriptive statistics were employed. This involved presenting data as means with standard deviations (SDs) for variables that followed a normal distribution, and as medians with interquartile ranges (IQRs) for other data. The statistical analysis was conducted using RStudio Version 4.4.0 and Microsoft Excel 365 (2021). The Student *t*-test compared two independent means, and Pearson correlation assessed the linear relationship between continuous variables. Significance was set at *p* < 0.05.

Levene’s test was employed to assess the homogeneity of variances, and the Kolmogorov–Smirnov test evaluated the normality of the data distribution. Given the non-uniformity of the data, as indicated by these tests, the Kruskal–Wallis test was conducted to compare HRV parameters across multiple groups. For post hoc analysis, Dunn’s test was used to identify specific group differences. The Chi-squared test was used to determine if there is a significant association between categorical variables. Confidence intervals (CIs) at the 95% level were calculated. 

The cardiology clinic conducted all of the examinations regarding HRV and echocardiography of the mothers. All participants gave informed consent in accordance with the principles set forth in the Declaration of Helsinki. Additionally, the study protocol was approved by the ethical review board at our institution. 

## 3. Results

### 3.1. Study Group Description

Our study included 70 patients in the case group and 70 patients in the control group, ensuring a balanced comparison between groups. The mean age of patients with HDP was 30.1 (4.2) years while in the control group the mean age was 28.2 (3.7) years (*p* = 0.008) suggesting that the patients in the HPD group tend to be older compared to those in the control group. This indicates that age may be a contributing factor to hypertensive disorders in pregnancy.

The difference in gestational age between the two groups with a mean of 27.3 (3.7) weeks and 26.7 (3.9) weeks is not statistically significant (*p* = 0.357), suggesting that the timing of pregnancy progression is similar between mothers with hypertensive disorders and those in the control group.

The mean of both SBP and DBP was significantly higher in the HPD group compared to the control group, given the nature of the condition (*p* < 0.001). The difference in heart rate between the two groups is not statistically significant (*p* = 0.1148), indicating that heart rate may not be markedly affected by hypertensive disorders in pregnancy. Moreover, the two groups were comparable in terms of body mass index and hemoglobin concentration (Table 1).

### 3.2. HRV Analysis

The comparison of HRV parameters between the HDP group and the control group reveals significant differences in all measured parameters. The hypertensive disorder group shows reduced HRV, lower parasympathetic activity, and altered autonomic regulation compared to the control group. In the time domain parameters, SDNN, reflecting overall HRV, is significantly reduced (*p* < 0.001). The RMSSD, indicating short-term variations in heart rate, is significantly lower (*p* < 0.001). The pNN50 is significantly decreased in the HPD group (*p* < 0.001). In the frequency domain parameters, TP, reflecting overall autonomic activity, is significantly lower (*p* < 0.001). The HF component, indicating parasympathetic activity, is significantly reduced (*p* < 0.001). The LF component, representing both sympathetic and parasympathetic activity, is significantly lower (*p* < 0.001) in hypertensive pregnant women. Finally, the LF/HF ratio is significantly higher in the HPD patients indicating a dominance of sympathetic over parasympathetic activity (*p* < 0.001). These findings suggest that hypertensive disorders during pregnancy are associated with significant changes in autonomic function (Table 2).

**Figure 1:**
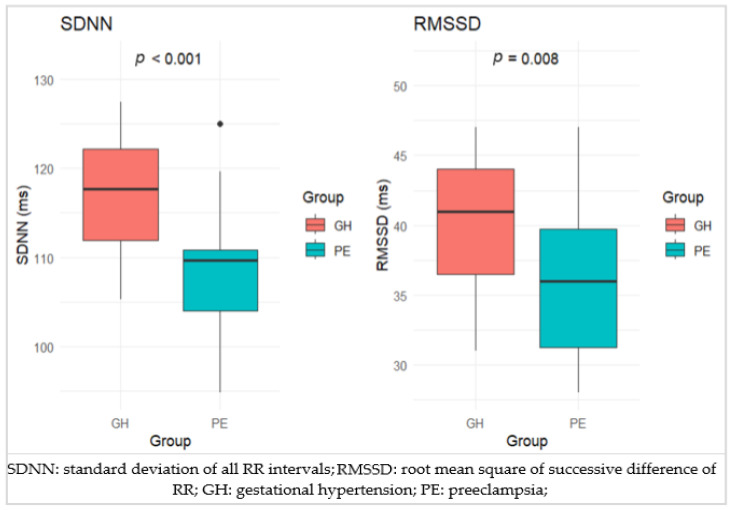
Comparison of SDNN and RMSSD between GH and PE.

Regarding the HRV parameters within the HPD subgroups, there was no statistically significant difference observed for the NN parameter among the groups GH (median = 936, IQR = 83.5), PE (median = 931, IQR = 61.25), and SPE (median = 907, IQR = 62) (*p* = 0.589). Significant differences were found in SDNN values across the groups GH (median = 117.7, IQR = 10.25), PE (median = 109.7, IQR = 6.82), and SPE (median = 117.2, IQR = 7.7) (*p* < 0.001). The RMSSD parameter exhibited significant variation between GH (median = 41, IQR = 7.5), PE (median = 36, IQR = 8.5), and SPE (median = 42, IQR = 8) (*p* = 0.008). No significant differences were observed in pNN50 values among GH (median = 12.9, IQR = 3.65), PE (median = 11.9, IQR = 3.05), and SPE (median = 12, IQR = 4.9) (*p* = 0.158), in TP values between GH (median = 2905, IQR = 524), PE (median = 2728, IQR = 407), and SPE (median = 3111, IQR = 527) (*p* = 0.217). There was no significant difference in HF values among GH (median = 403, IQR = 102.5), PE (median = 355, IQR = 94.5), and SPE (median = 352, IQR = 56) (*p* = 0.13). Similarly, no significant differences were found in LF values between GH (median = 931, IQR = 256), PE (median = 938, IQR = 264.5), and SPE (median = 1027, IQR = 191) (*p* = 0.566). The LF/HF ratio did not show significant differences among GH (median = 2.38, IQR = 0.9), PE (median = 2.71, IQR = 0.93), and SPE (median = 2.87, IQR = 0.65) (*p* = 0.102). Overall, significant differences were observed in SDNN and RMSSD parameters among the three groups. However, NN, pNN50, TP, HF, LF, and LF/HF parameters did not show significant differences across these groups. These findings suggest specific variability in ANS parameters (SDNN and RMSSD) but consistent values in other parameters across the groups and suggest that while overall autonomic regulation (as reflected by SDNN and RMSSD) differs among groups, the sympathovagal balance (as reflected by LF/HF) remains consistent. Further analysis shows a significant difference between the GH group and PE group regarding SDNN and RMSSD parameters (Z-value = 3.93, *p* = 0.0001 and Z = 2.85, *p* = 0.008), indicating that SDNN and RMSSD values are significantly higher in the GH group compared to the PE group, which suggest that patients with GH exhibit greater overall variability in their heart rate intervals, indicating better overall ANS function when compared to preeclamptic pregnant women (Table 3, Figure 1).

### 3.3. Cardiac Performance Analysis

**Figure 2:**
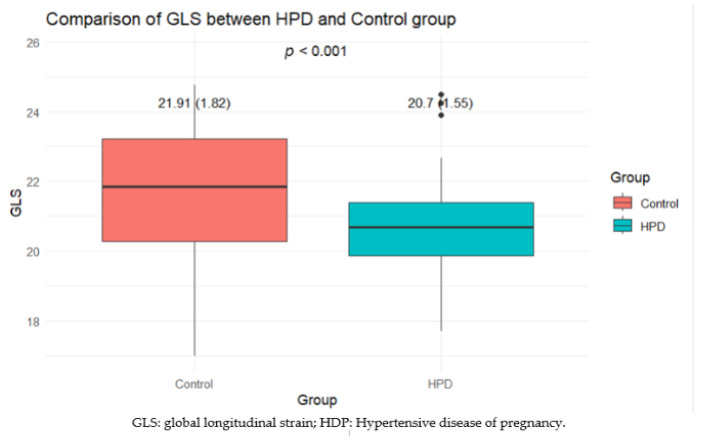
Comparison of GLS (%) between HPD and control group.

The mean GLS for the control group (21.91 (1.82)) is higher than that for the HPD group (20.7 (1.55)) (*p* < 0.001). This result suggests that patients with HPD have, on average, worse GLS values compared to those in the control group. It may indicate a greater impairment in myocardial function in the context of hypertensive disorders during pregnancy, highlighting potential cardiovascular differences that may be clinically relevant in this population (Figure 2).

**Figure 3:**
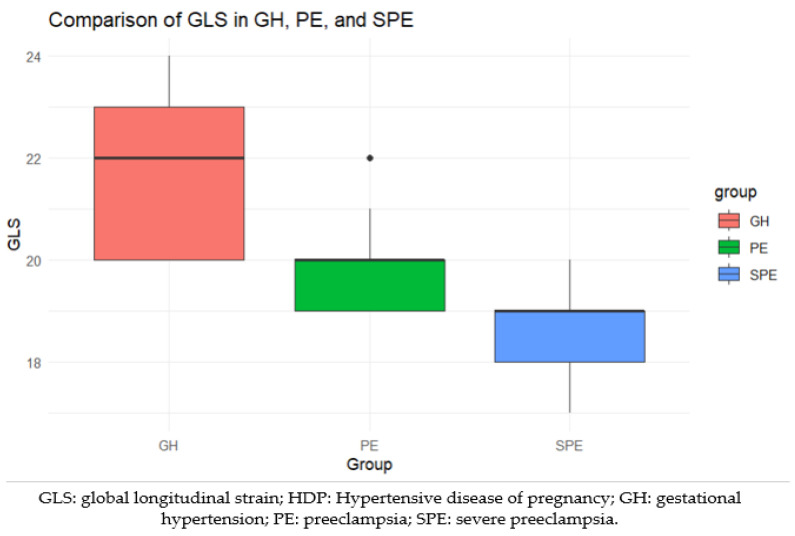
Comparison of GLS (%) between HPD groups.

There is a statistically significant difference among the three groups (GH, PE, and SPE) with respect to GLS (Chi-squared: 35.51, Degrees of Freedom: 2, *p* < 0.001). There are lower GLS values in preeclamptic patients compared to pregnancy-induced hypertension patients. Moreover, the most reduced values of the GLS are present in the SPE group (Figure 3).

**Figure 4:**
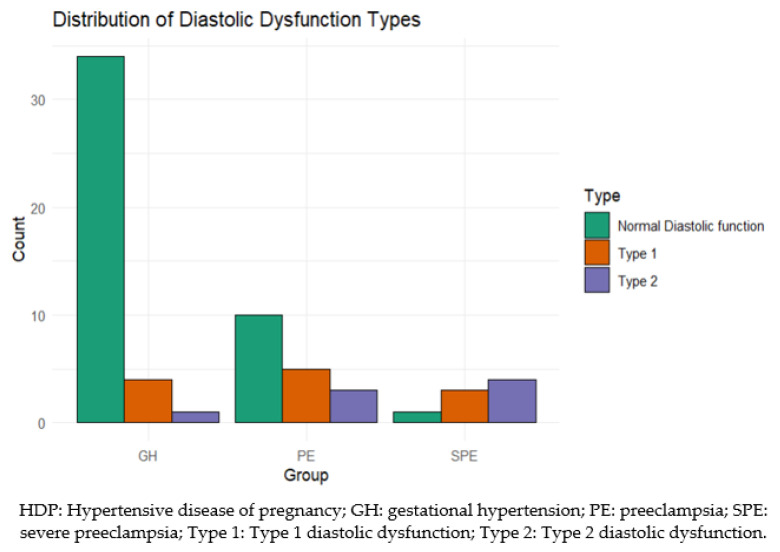
Comparison of diastolic function between HPD groups.

In women from the control group, no diastolic dysfunction was found. When analyzing the presence of diastolic dysfunction in patients with HPD, we found that 21.43% of patients presented type 1 diastolic dysfunction and 11.43% type 2 diastolic dysfunction, while no patient had type 3 or type 4 diastolic dysfunction. There is a statistically significant difference in the distribution of normal diastolic function, type 1, and type 2 across the three groups (X-squared: 20.13, df:4, *p* < 0.001). The statistically significant difference indicates that the prevalence of diastolic dysfunction types varies significantly among the groups GH, PE, and SPE. For example, the SPE group shows a higher prevalence within the group of type 1 (residual = 1.1538) and type 2 (residual = 1.6923) diastolic dysfunction compared to the other groups. Women with severe forms of PE more often exhibit diastolic dysfunction (Figure 4).

## 4. Discussion

The comparison of HRV parameters between the HDP group and the control group revealed significant differences in all measured parameters. The HDP group exhibited reduced HRV, indicative of lower parasympathetic activity and altered autonomic regulation compared to the control group. Specifically, time domain parameters such as SDNN, which reflects overall HRV, were significantly reduced, indicating diminished variability in heart rate. RMSSD, which highlights short-term variations in heart rate, was also significantly lower, and the pNN50 was significantly decreased in the HDP group.

In the frequency domain, TP, representing overall autonomic activity, was significantly lower. The HF component, indicative of parasympathetic activity, and the LF component, which represents both sympathetic and parasympathetic activity, were significantly reduced. The LF/HF ratio was significantly higher in the HDP patients, suggesting a dominance of sympathetic over parasympathetic activity. These findings underscore that hypertensive disorders during pregnancy are associated with significant autonomic dysfunction.

Within the HDP subgroups, there were no statistically significant differences in NN, pNN50, TP, HF, and LF values among the groups. The LF/HF ratio did not show significant differences across the subgroups. The significant difference in SDNN and RMSSD values between the GH and PE groups indicates better overall autonomic function in the GH group compared to the PE group.

Yang et al. [25] found that normotensive pregnant women exhibited a lower HF value, but higher LF/HF compared to nonpregnant women, suggesting an increase in sympathetic regulation and a decrease in parasympathetic influence during normal pregnancy. Our study did not include a nonpregnant comparison group, but our normotensive pregnant control group exhibited higher HRV and parasympathetic activity compared to the HDP group. 

Both studies found that preeclamptic women had lower HF and higher LF/HF compared to normotensive pregnant women. Our study additionally found that the TP and LF were significantly lower in the HDP group, indicating a reduction in overall autonomic activity. Yang et al.’s study [25] highlighted that preeclamptic pregnancy is associated with enhanced sympathetic regulation and further attenuated parasympathetic influence compared to both normotensive pregnant and nonpregnant groups. Our results align with this finding, as the HDP group showed reduced HRV, lower parasympathetic activity, and increased sympathetic dominance.

Our study and the Moors et al. review [26] found significant differences in HRV parameters between women with HDP and normotensive pregnant controls. However, there are some key differences and consistencies in the findings of our study compared to the range of studies reviewed by Moors et al. Our study identified a significant reduction in overall HRV in the HDP group compared to controls. Similarly, the Moors et al. review reported decreased overall HRV, particularly in PE, as evidenced by lower SDNN and HRV triangular index. Both our findings and those reviewed by Moors et al. [26] indicate sympathetic overdrive and parasympathetic withdrawal in HDP, suggesting an imbalance in autonomic regulation.

Our study showed significant reductions in TP, HF, and LF components, along with an increased LF/HF ratio in the HDP group, indicating elevated sympathetic activity and reduced parasympathetic activity. Moors et al. [26] found varied results across different studies, with some showing no difference, increases, or decreases in LF and HF components in PE compared to normotensive controls. However, the trend of an increased LF/HF ratio in PE reported in several studies aligns with our findings of sympathetic dominance.

Our study further stratified the HDP group into GH, PE, and SPE. Significant differences in SDNN and RMSSD were observed among these subgroups, with GH showing better autonomic function compared to PE. Moors et al. reviewed studies that also indicated variability within HDP subgroups, noting differences in autonomic regulation between early-onset and late-onset PE.

Both our study and the Moors et al. review underscore the significance of autonomic dysfunction in HDP. The consistent finding of elevated sympathetic tone and reduced parasympathetic activity in HDP highlights the role of autonomic imbalance in these conditions. Despite some variability in specific HRV parameters across different studies, the overall trend supports the use of HRV as a valuable tool for assessing autonomic regulation in pregnant women, with potential implications for predicting and managing HDP. 

A primary finding in our study is the trend toward increased sympathetic tone and decreased parasympathetic tone in HDP. This finding is consistent with other studies in the literature [27,28]. Increased sympathetic activity can elevate peripheral resistance, subsequently raising BP. 

Our study demonstrated significantly lower GLS values in the HDP group compared to controls, with the most reduced values in SPE. This difference in GLS highlights a greater impairment in myocardial function among pregnant women with hypertensive disorders, which may have important clinical implications [29,30].

Popescu et al. [29] similarly reported decreased GLS in pregnancies complicated by PE, highlighting its utility in detecting subclinical myocardial dysfunction and its association with long-term cardiac effects.

Both our study and the findings of Popescu et al. [29] underscore the significant cardiovascular risks associated with HDP. The consistent detection of reduced GLS and increased diastolic dysfunction in women with HDP highlights the need for early and accurate screening methods. These measures are crucial for predicting and managing preclinical cardiac dysfunction and reducing long-term cardiovascular morbidity and mortality in this population. This highlights potential cardiovascular differences that may be clinically relevant in this population [30].

Our study found a significant prevalence of diastolic dysfunction in the HDP group, particularly in SPE, with type 1 and type 2 diastolic dysfunction being the most common.

This suggests that the severity of PE correlates with the degree of diastolic dysfunction, emphasizing the impact of severe hypertensive conditions on cardiac function during pregnancy.

Muthyala et al. [31] reported similar findings regarding diastolic dysfunction. These findings align with our results, indicating a higher prevalence and severity of diastolic dysfunction in SPE.

Popescu et al. [29] noted that diastolic dysfunction is detectable in preeclamptic pregnancies and serves as an independent predictor of complications in women with chronic hypertension, aligning with our observations of higher diastolic dysfunction prevalence in SPE.

Overall, these findings emphasize the significant impact of hypertensive disorders on autonomic and cardiac function during pregnancy. The altered HRV parameters and reduced GLS in the HDP group suggest that autonomic dysfunction and myocardial impairment are prominent features of these conditions, necessitating careful monitoring and management to mitigate associated risks [18,26,32].

Our study has a few limitations. Extended ECG recordings are susceptible to noise and interference. Unlike short-term recordings, extended monitoring occurs in less controlled environments, which can pose challenges in maintaining signal quality. Additionally, measurements were taken at a single time point during pregnancy, which may not capture the dynamic changes in autonomic and cardiac function that occur throughout pregnancy and the postpartum period. Despite these limitations, ambulatory monitoring offers the advantage of recording parameter values over a longer duration, providing a more comprehensive evaluation of HRV and autonomic function. Future research can address these gaps.

## 5. Conclusions

In conclusion, our study highlights the impact of hypertensive disorders during pregnancy on both autonomic regulation and cardiac function. HRV and GLS are valuable non-invasive tools for detecting autonomic dysfunction and cardiac performance impairments in pregnant women with hypertensive disorders. Our findings indicate that autonomic and cardiac dysfunctions are prevalent in HDP, with significant implications for cardiovascular health and potential clinical management. The noted decrease in the sympathovagal balance highlights the need to address autonomic dysfunction in this group. Our study supports the hypothesis that HDP is characterized by sympathetic overdrive coupled with parasympathetic withdrawal.

## Figures and Tables

**Table 1:** Comparison between HDP group and control group.

Parameter	HDP	Control Group	*p*-Value (*p*)
MA (years)	30.1 (4.2)	28.2 (3.7)	0.008
GA (weeks)	27.3 (3.7)	26.7 (3.9)	0.357
SBP (mmHg)	156.9 (9.6)	122.4 (8.4)	<0.001
DBP (mmHg)	100.9 (10.6)	78.1 (5.5)	<0.001
HR (b/min)	88.9 (10.3)	86 (11.3)	0.1148
Hb (g/L)	12 (0.6)	11.8 (0.7)	0.157
BMI (kg/mp)	29 (3.8)	28.1 (4.3)	0.188

HDP: hypertensive disease of pregnancy; MA: mother age; GA: gestational age; SBP: systolic blood pressure; DBP: diastolic blood pressure; HR: heart rate; Hb: hemoglobin; BMI: body mass index.

**Table 2:** HRV parameters in HDP group and in control group.

Parameter	HDP Group	Control Group	*p*-Value (*p*)
SDNN (ms)	113.85 (14.82)	132.9 (9.61)	*p* < 0.001
RMSSD (ms)	39.25 (5.10)	59.75 (7.37)	
pNN50 (%)	12.88 (2.46)	20.38 (2.46)	
TP (ms^2^)	2855 (321.36)	3098.94 (188.91)	
HF (ms^2^)	394.17 (60.47)	536.2 (68.94)	
LF (ms^2^)	956.7 (155.10)	1086.08 (60.07)	
LF/HF	2.49 (0.59)	2.05 (0.23)	

HDP: hypertensive disease of pregnancy; SDNN: standard deviation of all RR intervals; RMSSD: root mean square of successive difference in RR; pNN50 (%): percent of successive RR differences > 50 ms; TP: total power; HF: high-frequency power; LF: low-frequency power; LF/HF: low-frequency/high-frequency ratio.

**Table 3:** HRV parameters across the different groups (GH, PE, SPE).

Parameter	GH Median (IQR)	PE Median (IQR)	SPE Median (IQR)	Chi-Squared	*p*-Value (*p*)
NN (ms)	936 (83.5)	931 (61.25)	907 (62)	1.05	*p* = 0.589
SDNN (ms)	117.7 (10.25)	109.7 (6.82)	117.2 (7.7)	17.68	*p* < 0.001
RMSSD (ms)	41 (7.5)	36 (8.5)	42 (8)	9.77	*p* = 0.008
pNN50 (%)	12.9 (3.65)	11.9 (3.05)	12 (4.9)	3.69	*p* = 0.158
TP (ms^2^)	2905 (524)	2728 (407)	3111 (527)	3.05	*p* = 0.217
HF (ms^2^)	403 (102.5)	355 (94.5)	352 (56)	4.08	*p* = 0.13
LF (ms^2^)	931 (256)	938 (264.5)	1027 (191)	1.13	*p* = 0.566
LF/HF	2.38 (0.9)	2.71 (0.93)	2.87 (0.65)	4.56	*p* = 0.102

SDNN: standard deviation of all RR intervals; RMSSD: root mean square of successive difference in RR; pNN50 (%): percent of successive RR differences > 50 ms; TP: total power; HF: high-frequency power; LF: low-frequency power; LF/HF: low-frequency/high-frequency ratio; IQR: interquartile range; GH: gestational hypertension; PE: preeclampsia; SPE: severe preeclampsia.

## Data Availability

The data presented in this study are available within the article. Further inquiries can be directed to the corresponding author at adrian.apostol@umft.ro.
